# Management of a pediatric patient with dental anomalies and its effect on psychosocial status: a case report

**DOI:** 10.3389/fdmed.2024.1502195

**Published:** 2025-01-10

**Authors:** Wesam Damanhouri, Kholoud Moussa, Joudi Bathallath, Zohour Alsomali, Abeer Bakor, Moaz Attar

**Affiliations:** ^1^ Pediatric Dentistry Unit, Dental Center, King Fahad General Hospital, Jeddah, Saudi Arabia; ^2^ Oral, and Maxillofacial Division, Dental Center, King Fahad General Hospital, Jeddah, Saudi Arabia; ^3^ Deanship of Graduate Studies and Research, Effat University, Jeddah, Saudi Arabia; ^4^ Pediatric Dentistry Unit, Dental Center, King Fahad General Hospital, Jeddah, Saudi Arabia; ^5^ Pediatric Dental Department, Faculty of Dentistry, King Abdulaziz University, Jeddah, Saudi Arabia

**Keywords:** supernumerary, case report, fusion, dental anomaly, psychosocial

## Abstract

Managing multiple dental anomalies in the anterior region of the dental arch presents unique and complex challenges. This case report describes using a multidisciplinary approach to manage a child with not only a fused tooth with a supernumerary but also the presence of other supernumerary teeth in the same place. In this case, deviation from the standard management of a fused permanent anterior tooth, an anchor for aesthetics, by extracting it because of crown root complexity and allowing a supernumerary to erupt into its place made the difference in the successful outcome. However, this condition required multidisciplinary management that necessitated the involvement of the psychiatric department. Continuous assessment of the patient's psychosocial function using the Social Avoidance and Distress Scale (SADS) allowed appropriate guidance to manage the patient's behavior. This case report added a different perspective on managing a fused tooth with a supernumerary to the literature. It showed how dental aesthetics can have an adverse effect on children's psychology.

## 1 Introduction

Dental anomalies can be defined as developmental tooth defects that are clinically heterogeneous in their phenotypes. In addition to genetic factors, dental anomalies originate from disturbances during tooth development (1). These disturbances can produce changes in the number of teeth (agenesis/supernumerary teeth), size (microdontia, macrodontia, taurodontism, and fusion), morphology, dental tissue composition, structural and environmental defects, hereditary sequence, and time of eruption (2). A supernumerary tooth is among those anomalies that can occur in both primary and permanent dentition separated from or fused to a normal tooth. However, its abnormal shape and large size require distinctive dental treatment, such as sectioning, orthodontic treatment, root canal treatment, or extraction (3–5). Studies have shown that supernumeraries occur in 76%–86% of cases, double supernumeraries in 12%–23%, and multiple supernumeraries in <1% of cases (6). Other studies have reported that the prevalence of tooth fusion ranges from 0.2% to 2.5% among different ethnicities (7–9). However, permanent and supernumerary tooth fusion is uncommon compared to primary and supernumerary tooth fusion. It has been shown that permanent and supernumerary tooth fusion is 0.1% and generally involves the maxillary anterior teeth (10).

Extant studies have reported various clinical challenges, including aesthetic difficulties, functional issues, tooth misalignment, and psychological problems, which are associated with a supernumerary fused to permanent teeth (1, 3, 11). One study used three scales to assess the psychological status of children who have problems with dental aesthetics: PIDAQ (Psychosocial Effect of Dental Aesthetics Questionnaire), MPS (Multidimensional Perfectionism Scale), and RSS (Rosenberg Self-esteem Scale). They concluded that there is a correlation between decent dental appearance and a child's improved self-esteem (12). A very recent study found that low self-esteem can be predicted in orthodontic patients who have dental aesthetic problems using different psychosocial scales, including the PIDAQ (13). However, none of those studies used other scales concerning social avoidance or social distress, like the Social Avoidance and Distress Scale (SADS).

Managing multiple dental anomalies often requires a multidisciplinary approach to achieve satisfactory outcomes. Therefore, supernumerary teeth can necessitate significant alterations in treatment to address functional and aesthetic concerns (14, 15). This case report describes the multidisciplinary approach used with a pediatric dental patient with a supernumerary tooth fused to the permanent central incisor. The objective was to restore oral function and aesthetic appearance by first managing the fused maxillary central incisors with a suitable treatment plan and measuring the aesthetic effect on the patient's psychosocial status.

## 2 Patient information

**Figure 1:**
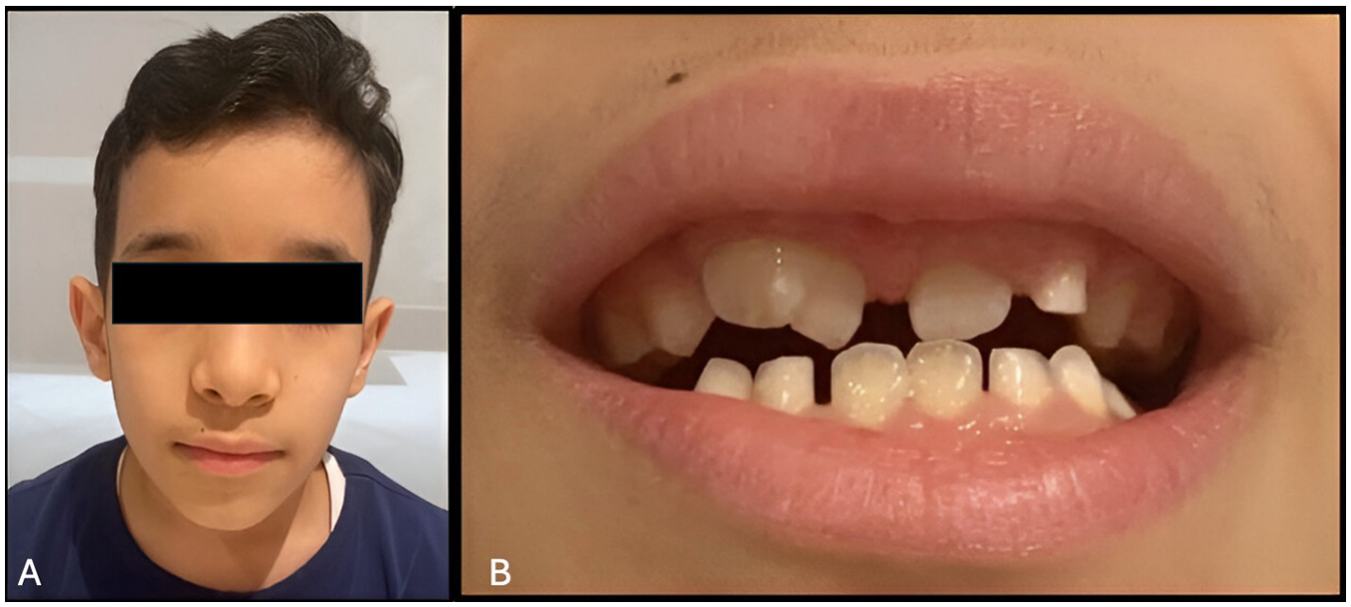
**(A)** Facial profile of the patient on the first visit. **(B)** Intra-oral picture shows a large right maxillary incisor.

A 10-year-old boy presented to King Fahad Hospital in Jeddah (KFHJ) Pediatric Dental Clinic with his mother because of a previous recommendation from a pediatric consultant. The chief complaint was: “My kid's smile is ugly, and he's been bullied at school because of his appearance” (Figure 1A). The patient's medical history was insignificant. Further, no history of a similar condition among family members was reported. The gestational period was unrelated to any insult, and he has no known allergies. His past dental history revealed no pain, and he had never needed dental fillings.

## 3 Clinical findings

Upon extra-oral examination, it was noticed that the patient always wears his surgical face mask and tries to hide his mouth when he speaks. When a question is directed to him, he looks at his mother and whispers his answers to her to convey the response; otherwise, everything is within normal limits. Intra-oral examination revealed good oral hygiene, sound teeth, and a large permanent maxillary right anterior tooth (#11) (Figure 1B).

## 4 Diagnostic assessment

**Figure 2:**
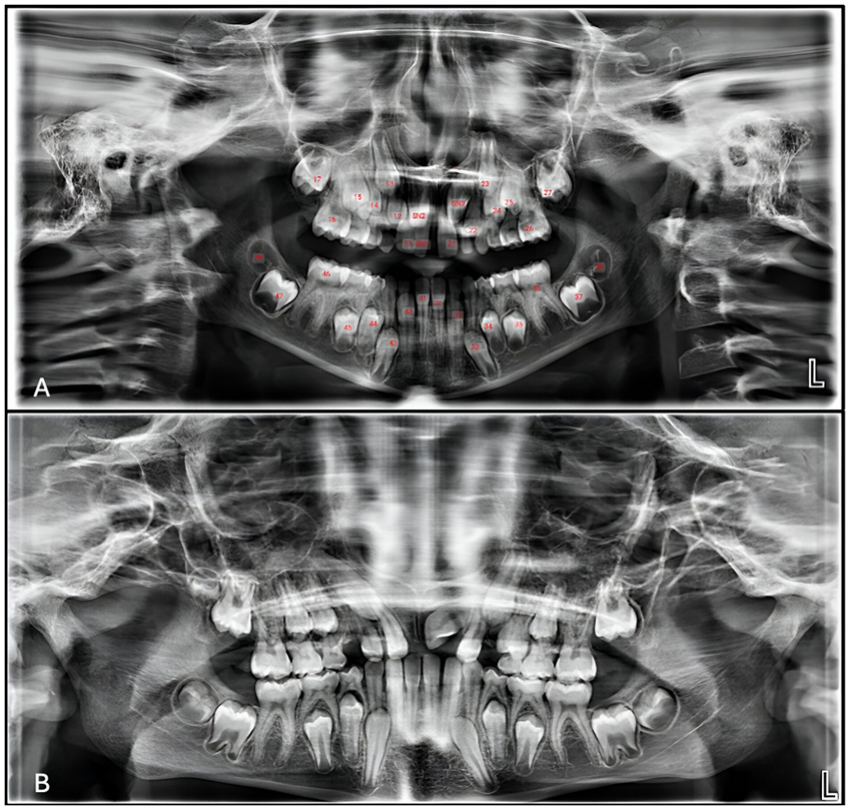
**(A)** Panoramic radiograph of tooth count shows multiple supernumeraries (SN) and right maxillary central incisor fused with SN1. **(B)** Panoramic radiograph revealing the redirection and eruption of SN2 and SN3.

It has been shown that tooth count is essential to differentiate between fusion and gemination. In this study, a panoramic radiograph showed a maxillary right central incisor (#11) fused with a supernumerary (SN1). Impacted supernumeraries (SN2, SN3) were also found, together with the absence of maxillary third molars' buds (Figures 2A,B). Cone-beam computed tomography (CBCT) confirmed the supernumeraries' position and SN3's proximity to the nasal floor.

## 5 Therapeutic intervention

**Figure 3:**
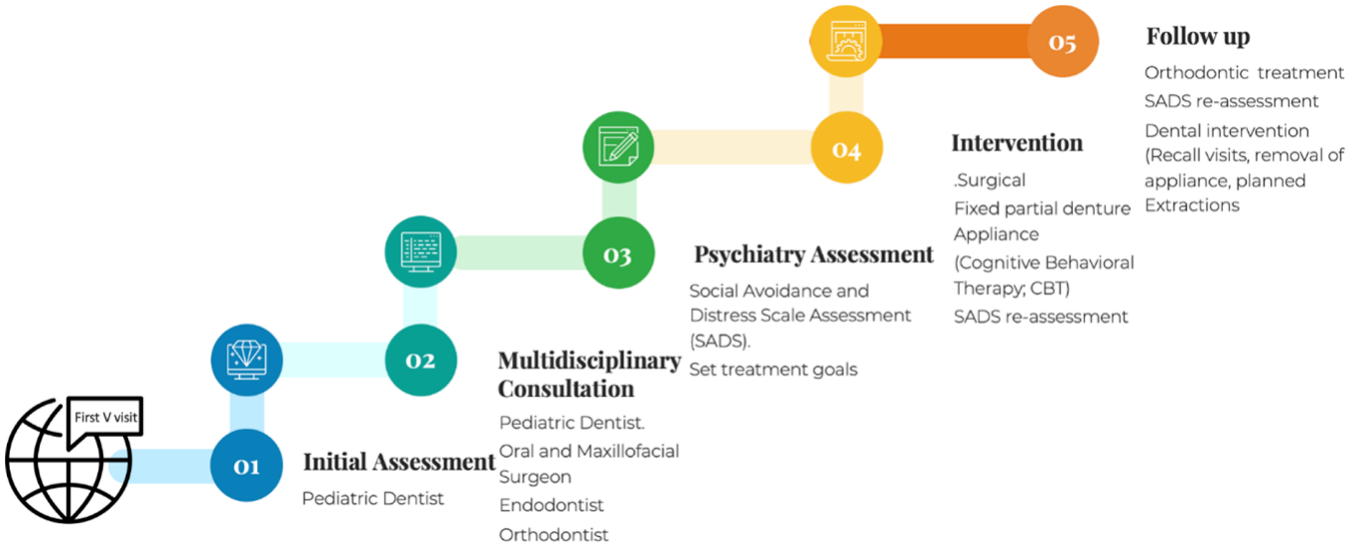
Shows the patient's therapeutic journey.

The combined management of the pediatric dentist, oral and maxillofacial surgeon, and psychiatrist gave the case a distinctive presentation. Figure 3 shows the patient's therapeutic journey step-by-step, including all of the stages of the treatment (Figure 3).

### 5.1 Dental assessment and management

With all given clinical findings, clinical consultations were performed with the orthodontic and endodontic consultants to discuss the case prognosis and best treatment plan. Consultation with endodontics was not promising; the fusion line was deep within the roots, so it was impossible to reshape or restore the tooth (#11) endodontically. Orthodontic consultation recommended extracting the affected tooth (#11) to allow the impacted supernumerary tooth to erupt in its place before the orthodontic treatment began. On the other hand, the oral and maxillofacial surgeon recommended a surgical extraction of the fused supernumerary teeth (fused tooth no. 11, impacted SN2, and SN3) to be performed under general anesthesia (GA) (Figures 2A,B).

Finally, the pediatric dentist's plan was to construct a partial denture to restore the patient's aesthetic appearance and oral function after the operation. Hence, the treatment plan selected was discussed with the mother, and a consent form was obtained. It is worth mentioning that the pediatric dentist who treated the child noticed the child's lack of self-confidence, shyness, and low self-esteem. As part of the usual pediatric dentist's practice, this observation was detected by observing the child's behavior of covering his mouth with his hand continuously while responding to the dentist's questions. Another notable behavior was that the patient hid behind his mother most of the time during the first visit. Further, his “crackling” voice was apparent, and he avoided eye contact. Therefore, referring the child to a psychologist was necessary to address the patient's lack of self-confidence and improve his communication skills.

**Figure 4:**
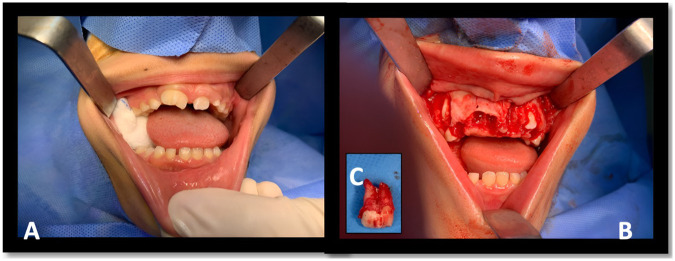
**(A)** Intra-oral view. **(B)** Flap exposure of SN. **(C)** The extracted fused tooth.

In the operating room under GA, the oral maxillo-facial surgeon created a flap to expose the supernumerary teeth, which had good shape and anatomical form (Figures 4A–C). As planned, tooth #11 fused to SN1 was extracted to allow SN2 to erupt into position. Unexpectedly, there was a high risk in extracting the underlying supernumerary tooth (SN3) because of its proximity to the nasal cavity and the risk of perforation (16). After a quick consultation with the pediatric dentist, the surgeon decided to extract tooth number # 21 and allow the supernumerary tooth (SN3) to erupt in its place. The surgical flap was closed using a 3/0 Vicryl suture.

### 5.2 Psychological assessment and management

Given the patient's lack of confidence and high anxiety, it became crucial to consult a clinical psychologist. The psychologist conducted an intake interview with the mother to assess the child's psychological health, understand his medical and psychological history, determine the diagnosis, and develop a treatment plan. Based on the interview, the psychologist used the Social Avoidance and Distress Scale (SADS) to assess whether the child has social avoidance or social distress. Those problems include escaping from others or avoiding talking to them/being with them and experiencing negative emotions, such as being upset or anxious. Watson and Friend developed this 28-item true/false questionnaire, which is used commonly with children at our hospital, in 1969. The questionnaire's Cronbach alpha was found to be 0.94, which shows good reliability (17). However, high SADS scores are related to increased levels of anxiety and low self-esteem. In this case report, initially the patient scored 25, indicating high social anxiety levels. The psychologist decided to schedule numerous Cognitive Behavioral Therapy (CBT) sessions for the child. As a result, the SADS score dropped to 10, suggesting average anxiety. To the authors' knowledge, no study has been found to use the SADS with a pediatric dental patient, as in our study.

## 6 Follow-up and outcomes follow-up

**Figure 5:**
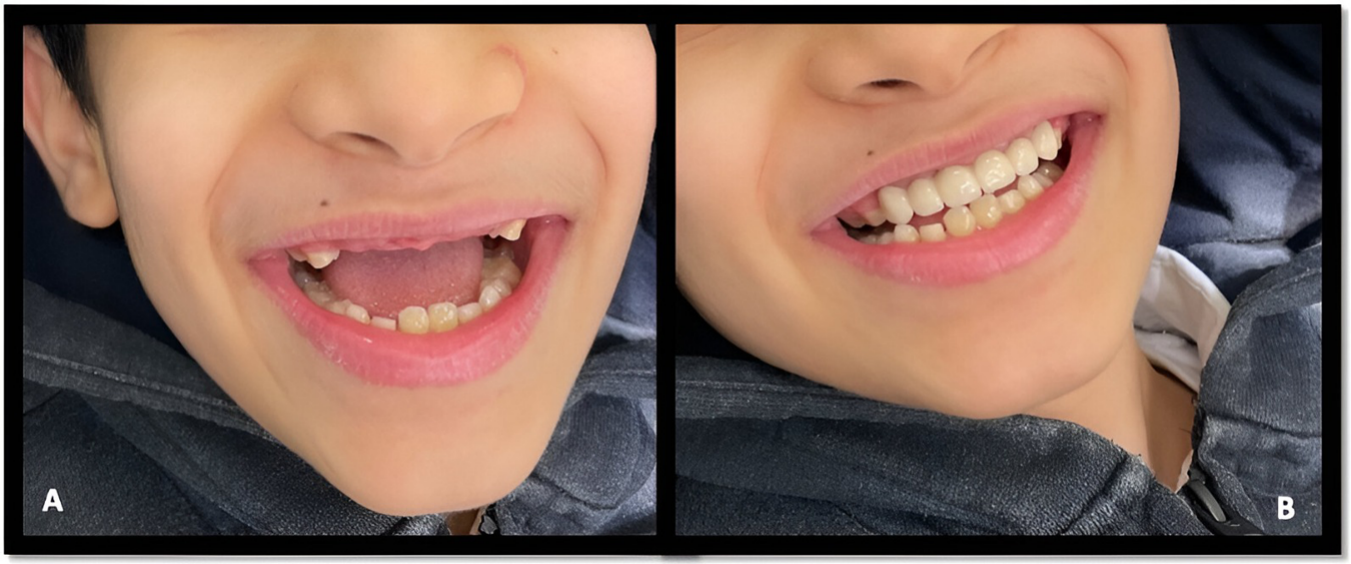
**(A)** Before the insertion of the fixed partial denture. **(B)** After the Insertion of the fixed partial denture.

Several weeks post-operatively, after the soft tissue had healed properly (Figure 5A), the consulting pediatric dentist constructed a fixed partial denture. The edentulous area caused a significant change in the patient's self-confidence after the appliance's insertion. As a result, the child was delighted with his new smile and he seemed more social and confident during follow up visits.

## 7 Discussion

The dental literature advocates different clinical approaches to managing fused/geminated teeth. Those approaches include orthodontic treatment, tooth extraction, crown size reduction, restoration, sectioning fused teeth, or no treatment (3, 7, 18). Each approach can be chosen according to several factors, including the degree of severity, the type of supernumerary tooth, the patient's age, and dental health overall.

In this case, the suggested management of dissecting the tooth and performing a root canal was not applicable. This was because of the difficulty sectioning the fused tooth, as the root canals joined longitudinally. Adjusting the crown size and shape without compromising the crown-root ratio was also impossible. When the fused teeth are attached down the gingival line, and the pulp chamber and root canals are connected, separation and root canal treatment are not feasible. This situation required the fused teeth to be extracted, which allowed the supernumerary tooth (SN2) to erupt. In contrast to other studies, extraction of anterior permanent tooth number #21 was mandatory because of the underlying supernumerary teeth's proximity to the nasal floor, which, in turn, could lead to potential complications upon their removal, such as nasal floor perforation (19, 20). A recent review found approximately 30 case reports of dental fusion, including permanent and supernumerary teeth. Most cases followed the conventional approach, which includes dental treatment with a surgical approach and orthodontic treatment (7).

On the other hand, any child can be a victim of bullying and feel anxious about their appearance, as research has shown that children with apparent dental abnormalities are highly vulnerable to repeated hostile actions on the part of their age mates or classmates (21). Nevertheless, using the SADS scale demonstrated strong validity and reliability in this case. Similarly, other studies, such as those of Watson and Friend (17), reported excellent internal consistency and good reliability (17). However, it has been shown that the SADS scale is used most frequently to determine social anxiety and social phobia (22). Our finding was consistent with that of Geist and Borecki's (23) study, which found that a high SADS score is associated with reduced self-confidence, less social affiliation, lower self-esteem, and decreased need for dominance (9).

The child's treatment plan integrated dental and psychological methods in this case report. This combined therapy is designed for rapid and effective results. In healthcare settings, the multidisciplinary approach is considered the most effective way to manage comprehensive cases, as in this case. In pediatric dentistry, multidisciplinary cooperation is taking place not only between different dental specialties but also between pediatric dentists and other specialties, such as pediatric physicians, pediatric surgeons, otolaryngologists, and, most importantly, psychologists/psychiatrists who specialize in treating children. In turn, this collaboration will affect healthcare service positively by applying the notion of holistic dentistry while minimizing the number of visits, particularly for complex cases (24).

In this case, the pediatric dentist constructed a removable aesthetic appliance to enhance the child's appearance, which affected his psychological well-being positively and, in turn, reduced his anxiety level. At the same time, the clinical psychologist used CBT to modify his distorted thinking and behavior and improve emotional regulation. Similarly, other studies have demonstrated that tooth color, shape, and alignment influence a patient's self-confidence and satisfaction significantly (25). Thus, it is not surprising that crowding of the teeth leads to adverse psychosocial effects, including poor self-esteem and social acceptance (26).

This collaborative care is one strength of this study. Another strength is the thorough follow-up in management between the pediatric dentist, oral and maxillofacial surgeon, and psychiatrist, which made a great difference in the child's self-confidence in a short period of time. One limitation is the lack of detailed psychosocial data that may help in presenting and, in turn, understanding psychological management in detail.

## 8 Conclusion

The decision on the best course of treatment should be made on a case-by-case basis, considering each patient's specific circumstances. Careful and individual diagnosis is crucial to manage such rare cases successfully. In this case, a proper understanding of tooth anatomy and multidisciplinary teamwork contributed to successful case management. However, a profound knowledge of the patient's psychosocial needs helped improve the outcome as well.

## Data Availability

The original contributions presented in the study are included in the article/Supplementary Material, further inquiries can be directed to the corresponding author.
